# Does the nature of solid feed influence rumen development? A comparative study of calves fed a high milk replacer with starter concentrate or alfalfa hay

**DOI:** 10.29374/2527-2179.bjvm009725

**Published:** 2026-01-19

**Authors:** Noelia Vazquez, Dellis dos Santos, Rody Artigas, Germán Antúnez, Nicolás Amaro, Mario López, Richard Mas, Cecilia Cajarville

**Affiliations:** 1 Departamento de Biociencias, Unidad de Anatomía- Facultad de Veterinaria, Montevideo, Uruguay.; 2 Departamento de Producción Animal y Salud de los Sistemas Productivos, Unidad de Mejora Animal- Facultad de Veterinaria, Montevideo, Uruguay.; 3 Departamento de Ciencias Veterinarias y Agrarias-CENUR-LN, Facultad de Veterinaria, Universidad de la República, EEMAC, Paysandú, Uruguay.; 4 Departamento de Producción Animal y Salud de los Sistemas Productivos, Facultad de Veterinaria, Universidad de la República, Libertad, San José, Uruguay.; 5 Veterinarian autonomus, Montevideo, Uruguay.; 6 Departamento de Producción Animal y Salud de los Sistemas Productivos, Facultad de Veterinaria, Universidad de la República, Libertad, San José, Uruguay. Actual adress: Sintesisnutricion S.L., Consulting services, Barcelona, Spain.

**Keywords:** calf, forestomach, papillae, bezerro, rúmen, papilas

## Abstract

This study evaluated the effects of two milk replacer-based diets, one supplemented with starter feed and the other with alfalfa hay, on rumen development in preweaned Holstein calves. Twenty newborn male calves were fed 8 L/day of milk replacer plus either starter feed (group A) or alfalfa hay (group B) *ad libitum* for 10 weeks. Rumen morphometry and histological features were assessed via gross anatomical evaluation, histology, and scanning electron microscopy. The calves in group A consumed significantly more solid feed and exhibited greater papillae development, with higher papillae height, width, and density per cm^2^, than those in group B. Moreover, the surface enlargement factor was consistently greater in group A across ruminal regions, except in the caudodorsal blind sac (*saccus cecus caudodorsalis*; *p* < 0.05). Empty rumen weight was also higher in starter-fed calves, suggesting enhanced mucosal development and absorptive capacity. Notably, keratin layer thickness did not explain the observed differences between the groups. Despite similar final body weights and growth, calves supplemented with starter feed showed greater rumen papillae development and absorptive surface area than those supplemented with alfalfa hay. These findings suggest that starter feeding during high milk allowance promotes epithelial maturation and enhances post-weaning absorption of fermentation products.

## Resumo

Este estudo avaliou os efeitos de duas dietas à base de sucedâneo do leite — uma suplementada com concentrado inicial (starter feed) e a outra com feno de alfafa — sobre o desenvolvimento do rúmen em bezerros Holandeses não desmamados. Vinte bezerros machos recém-nascidos receberam 8 L/dia de sucedâneo do leite mais concentrado inicial *ad libitum* (Grupo A) ou feno de alfafa *ad libitum* (Grupo B) durante 10 semanas. A morfometria e as características histológicas do rúmen foram avaliadas por meio de exame anatômico macroscópico, histologia e microscopia eletrônica de varredura. Os bezerros do Grupo A consumiram significativamente mais alimento sólido e apresentaram maior desenvolvimento papilar, com maior altura, largura e densidade de papilas por cm^2^. O fator de ampliação da superfície (SEF) foi consistentemente superior no Grupo A em todas as regiões ruminais, exceto no saco-cego caudodorsal (*saccus cecus caudodorsalis*) (*p* < 0,05). O peso vazio do rúmen também foi maior nos bezerros alimentados com concentrado, sugerindo maior desenvolvimento da mucosa e capacidade absortiva. Em contraste, a espessura da camada de queratina não explicou as diferenças observadas entre os grupos. Apesar do peso corporal final e do crescimento semelhantes, os bezerros suplementados com concentrado inicial apresentaram maior desenvolvimento papilar ruminal e área de superfície absortiva. Esses resultados indicam que a oferta de concentrado durante programas de alta ingestão de leite promove a maturação epitelial e pode potencializar a absorção de produtos de fermentação no período pós-desmame.

## Introduction

Accelerated rearing is an alternative to conventional calf-raising systems that aims to mimic the natural milk intake of calves raised by their mothers. This approach involves doubling or even tripling the typical milk allowance in conventional systems by supplying approximately 20% of body weight (BW) in whole milk or milk replacer (MR) containing 24-26% crude protein, along with *ad libitum* access to calf starter until weaning (Lagger, 2010). Traditional milk feeding (approximately 10% BW during the initial weeks of life) does not meet the nutritional demands of Holstein calves, potentially limiting their growth potential (Vieira et al., 2008).

By increasing energy and protein intake, accelerated rearing improves body development, reduces rearing costs, increases weaning weight, promotes early onset of puberty, and enhances mammary parenchymal development (Brown et al., 2005a, 2005b).

In calf-rearing systems, forage is often included in diet to promote rumen development via volatile fatty acid (VFA) production. These VFAs stimulate muscular layer growth and maintain epithelial health by reducing keratinization and increasing papillae development (Diao et al., 2019; Tamate et al., 1962). Notably, when hay and starter feed are offered simultaneously, calves prefer starter feed (Webb et al., 2014).

Morphological and physiological adaptations of the ruminant digestive system are strongly influenced by diet. Even slight modifications in early life feeding and nutrition profoundly impact rumen development, leading to lasting consequences for growth performance, health status, and future milk production in adult cattle (Diao et al., 2019). Although ruminal papillae development during fetal life does not differ between species (Scocco et al., 2007), postnatal development is shaped by the type, quantity, and quality of feed, directly affecting the VFA absorptive capacity. The extent of papillae development is determined by the feed energy content and VFA production rate (Brownlee, 1956; Weigand et al., 1975).

The stratified squamous epithelium of the rumen (ruminal papillae) is the primary VFA absorption site. Its structure undergoes significant modifications, particularly epithelial cell hyperplasia, with the initiation of solid feed consumption. Physical characteristics of feed, such as particle size, also influence epithelial development (Diao et al., 2019).

Increased dietary starter feed does not appear to influence ruminal musculature development but enhances papillae density and height (Flatt et al., 1959; Rickard & Ternouth, 1965; Stobo et al., 1966). However, excessive starter feed intake leads to the rapid accumulation of fermentation end-products, resulting in a lower ruminal pH (Beharka et al., 1998), reduced rumen motility (Nocek, 1997; Owens et al., 1993), excessive papillae growth with keratinization (Nocek & Kesler, 1980), and decreased VFA absorption (Hinders & Owen, 1965). Therefore, adding fiber to high-concentrate diets has been hypothesized to mitigate ruminal health issues (Cozzi et al., 2002; Suárez et al., 2006, 2007).

This established framework of ruminal adaptation, along with our previous finding of no substantial difference in abomasal morphology between calves fed the two dietary supplements in the same experiment (Vazquez et al., 2025), suggests that the rumen is a critical organ impacted by the divergent effects of solid feed. Therefore, in this study, we hypothesized that Holstein calves fed a high-quality MR combined with starter feed exhibit greater papillae development than those fed a MR with alfalfa hay.

This study aimed to determine the effects of two MR-based diets, one supplemented with starter feed and the other with alfalfa hay, on rumen development. By determining which diet leads to greater papillae growth and a larger absorptive surface for nutrients and VFAs, this study provides key insights to improve ruminant life adaptation and enhance calf performance.

## Materials and methods

### Experimental design and animals

This study was conducted at the Instituto de Producción Animal, Facultad de Veterinaria, Universidad de la República (UdelaR, route 1, km 42, Libertad, San José, Uruguay; 34°40’40”S, 56°32’13”W). During the study, the average ambient temperature was 22.1 °C, and average relative humidity was 66.2%, according to data from the Uruguayan Institute of Meteorology.

Twenty newborn male Holstein calves with adequate passive transfer of immunity (> 8.4% Brix) (Deelen et al., 2014) were included in this study. The calves were individually housed in 2 m × 1 m pens under controlled sanitary and environmental conditions. After one week of adaptation, all calves were fed 8 L/day of MR (12% dry matter [DM]) in two meals (08:30 and 16:30) at 38 ± 1 °C.

The animals were blocked by initial BW and week of birth and randomly assigned to one of the following two treatment groups:

- **Group A (CON)**: MR + starter concentrate *ad libitum* (average initial BW: 41.4 ± 2.2 kg).
- **Group B (ALF)**: MR + alfalfa hay *ad libitum* (average initial BW: 39.1 ± 4.2 kg).

A gradual weaning protocol was implemented from day 57 (week 8). During weeks 9 and 10, all calves were provided *ad libitum* access to alfalfa hay and starter feed and remained in their original pens.

All experimental procedures were reviewed and approved by the Animal Use Ethics Committee (Comisión de Ética de Uso de Animales; CEUA protocol Nº 685) of Facultad de Veterinaria, UdelaR.

### Feed analysis and intake measurements

Feedstuffs (Table 1) were analyzed for DM (Association of Official Analytical Chemists [AOAC] 934.01), ash (942.05), crude protein (955.04), and ether extract (920.39) following Association of Official Analytical Chemists (2000) procedures. Neutral and acid detergent fiber values were determined using the Van Soest method (Van Soest et al., 1991), with residual ash subtracted (Licitra et al., 1996). Metabolizable energy was estimated according to National Research Council (2001).

**Table 1:** Chemical Compositions of the Milk Replacer, Starter Feed, and Alfalfa Hay.

**Variables**	**Milk replacer**	**Starter feed**	**Alfalfa hay**
DM (%)	95.3	90	90.5
Crude Protein (% DM)	21.5	18	16
NDF (% DM)	−	17.9	40.4
ADF (% DM)	−	7.2	32.4
Ash (% DM)	5.6	5.6	7.2
Ether extract (EE) (% DM)	20	3.4	-
ME (Mcal/kg DM)^1^	4.58	3	1.96
Aflatoxins B1, B2, G1, G2 (ppb)*	-	< 5	-
DON (ppb)*	-	< 500	-
Zearalenone (ppb)*	-	< 50	-

The variables indicated with * correspond to values reported on the product label.

Dry matter (DM), Neutral Detergent Fiber (NDF), Acid Detergent Fiber (ADF), Deoxynivalenol (DON).

1 Metabolizable energy (ME) was calculated according to Drackley (2008) for milk replacer, and according to National Research Council (2001) for concentrate and alfalfa hay;

Lactose was estimated as: DM-(CP+EE+Ash).

Starter feed concentrate (per kg of DM) contained: 0.8-1.1% Ca, 0.7-0.9% P, 4.800 IU vitamin A, 600 IU vitamin D3, 120 mg vitamin E, 28.8 mg vitamin B1, 7.2 mg biotin, 5.760 mg Cu, 18.000 mg Mn, 0.228 mg I, 18.000 mg Zn, 0.062 mg Co, 0.115 mg Se, 16.800 mg Fe, and 30 mg of monensin.

Daily intake of MR with solid feed (starter or alfalfa hay) was calculated as the difference between the offered and refused amounts. BW was recorded weekly for 10 weeks. The proportion of forage in the total diet was calculated as the ratio of alfalfa hay intake to total solid feed intake.

### Anatomical and morphometric evaluation of the rumen

At the end of the experiment, all animals were humanely euthanized after 2-h fasting using a captive bolt device, followed by exsanguination via the jugular vein and carotid artery.

The rumen was dissected free of the peritoneum and weighed both full and empty. Empty rumen weight was determined after manual removal of contents, rinsing with running water, and draining for 10 min. All measurements were performed by the same operator to avoid bias.

Samples (approximately 3 cm^2^) were collected from six ruminal regions, namely the dorsal sac (DS), ventral sac (VS), atrium (A), recess (R), caudodorsal blind sac (CDBS), and caudoventral blind sac, and fixed in 10% formalin for histological analysis.

Papillae height, width, and density were measured from digital micrographs, and the surface enlargement factor (SEF) was calculated as follows (Equation 1):


$$\frac{SEF\;=\left(Number\;of\;papillae\;\times \;height\;\times \;width\right)\;+\;Basal\;area\text{​}}{Basal\;area\text{​}}$$ (1)

SEF was assumed to be 1 in regions without papillae (e.g., DS).

### Histology and scanning electron microscopy

For scanning electron microscopy, tissue samples (1 cm × 1 cm) were collected from each ruminal region of two randomly selected calves per treatment. The samples were rinsed, fixed in 4% paraformaldehyde, dehydrated through a graded ethanol series, critical-point dried, mounted on aluminum stubs, and gold-sputtered. Images were obtained using the Jeol JSM 5900 LV scanning electron microscope at the Facultad de Ciencias, UdelaR.

For histology, the tissue samples were processed at the Universidade Federal de Santa Maria (Brazil) via standard paraffin embedding, sectioned at 5 μm, and stained with Masson-Goldner trichrome. The slides were examined under the Olympus BX60 microscope (10×). Keratin layer thickness was measured at the apex of 10 papillae per region from one animal per treatment using Image ProExpress 6.0 (Media Cybernetics).

### Statistical analyses

Data distribution was verified using the Shapiro-Wilk test. Quantitative variables meeting the assumption of normality were compared between the groups using *t*-tests for independent samples.

BW, total feed intake, and BW gain were analyzed using a generalized linear mixed model in SAS (SAS Institute Inc., Cary, NC, USA). This model was selected because it allows the inclusion of both fixed and random effects, providing more accurate estimation of treatment effects while controlling for sources of random variation. Specifically, treatment was included as a fixed effect, block (defined by initial BW and week of birth) as a random effect, and initial BW as a covariate to adjust for potential individual differences at baseline.

Multiple comparisons among least-squares means were conducted using the Tukey-Kramer adjustment to control for type I error. Results are expressed as the least-squares means ± standard deviations, and statistical significance was set at *p* ≤ 0.05.

## Results

Throughout the study, MR intake was similar between the two groups (Table 2). However, over the entire experimental period up to week 10, the calves in group A consumed significantly more starter feed (*p* < 0.01), higher total amounts of solid feed and total DM (*p* < 0.01), and less alfalfa hay (*p* < 0.01) than those in group B. Notably, no significant differences in final BW, total BW gain over 10 weeks, and feed efficiency were observed between the two groups.

**Table 2:** Total Feed Intake and Performance Throughout the 10-Week Experiment.

**Item**	**Treatments** 1		**SEM**	***P*-value** 2
	**Alfalfa hay**	**Starter feed**		
**Feed ingestion (wk 1-10 of life)**				
Total MR intake, kg DM	46.4	46.7	0.52	0.915
Total starter DM intake, kg	14.4	37.8	3.11	< 0.01
Total alfalfa hay DM intake, kg	17.0	5.7	1.56	< 0.01
Total solid feed DM, kg DM^3^	31.2	43.3	3.41	< 0.01
Total DM intake, kg^4^	108.8	133.2	7.10	< 0.01
Proportion of forage, %^5^	54.5	13.2	2.76	< 0.01
Forage/Concentrate, %	1.18	0.15		< 0.01
**Performance (wk 1-10 of life)**				
Final BW, kg^6^	77.0	80.5	3.09	0.246
Total BW gained, kg ^6^	36.9	40.3	3.08	0.246
Gain-to-feed, %^7^	34.8	31.1	2.65	0.229

1 Treatments: The animals received 8 liters of milk replacer (MR) per day, free access to alfalfa hay or starter feed as solid feed before weaning (wk 8), and free access to alfalfa hay and starter feed from week 9 to 10 of the study.

2 Effect of treatment.

3 Total solid feed intake: sum of alfalfa hay and starter feed intake.

4 As the sum of dry matter (DM) intake from MR, alfalfa hay, and starter feed.

5 Forage intake as a proportion of the sum of concentrate and forage consumed.

6 Adjusted for gut fill similarly to Antúnez-Tort et al. (2023).

7 As the difference between final and initial body weight (BW) corrected for gut fill, divided by total feed intake.

The average empty rumen weight was higher in group A (1.99 kg) than in group B (1.54 kg), and this difference was statistically significant (*p* = 0.0007). The average full rumen weight was also higher in group A (9.68 kg) than in group B (9.64 kg); however, this difference was not statistically significant.

The full rumen accounted for 10.57% of BW in group A and 10.93% of BW in group B. Empty rumen weight as a percentage of BW was 2.18% in group A and 1.76% in group B. In both cases, the differences were not statistically significant (*p* = 0.958).

The vertical distance from VS to DS averaged 42.6 cm (±3.69) in group A and 40.3 cm (±5.08) in group B, with no significant difference between the groups (*p* = 0.131).

**Figure 1:**
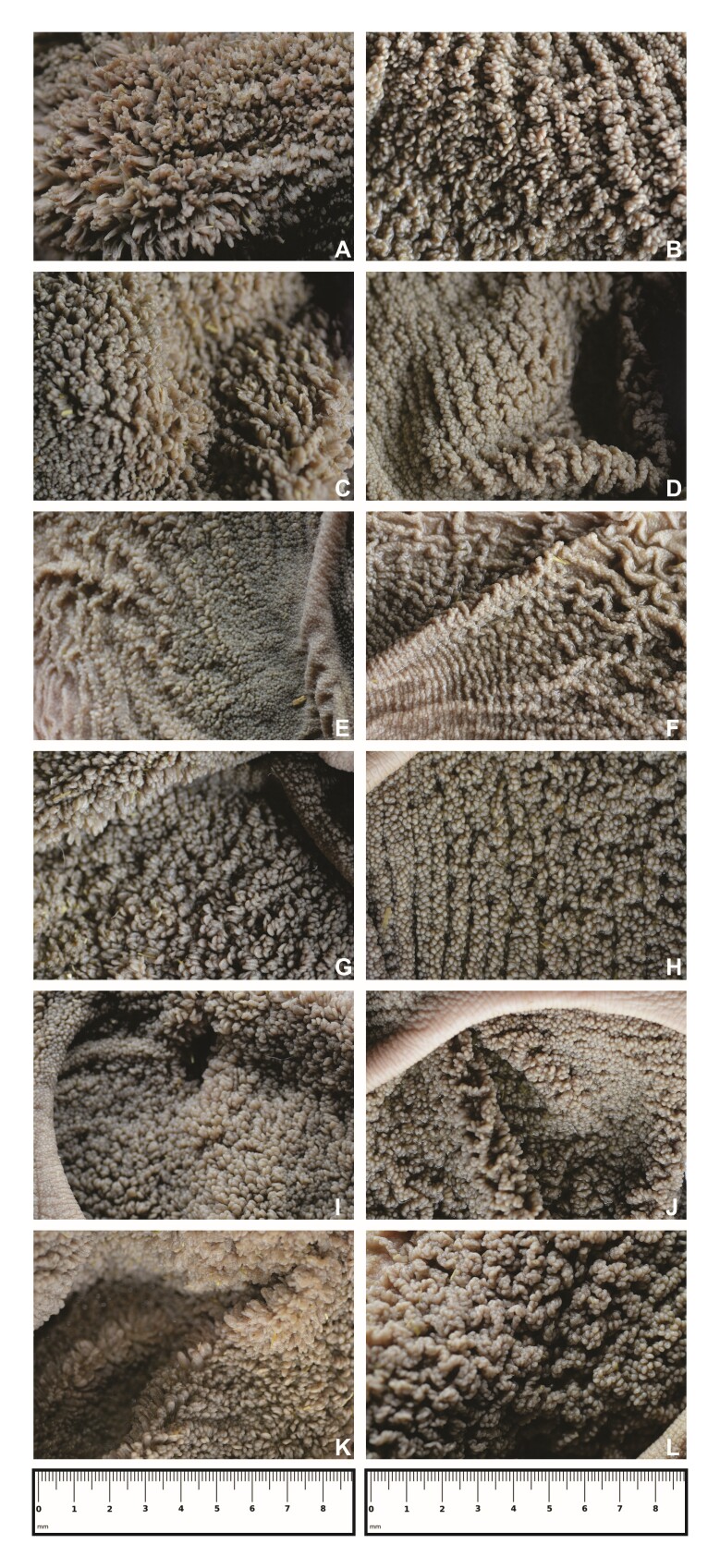
Macroscopic Images of the Ruminal Epithelium in Different Chambers Showing Papillae Development. Left, group A; right, group B. (A and B) Atrium; (C and D) Recess; (E and F) Dorsal sac; (G and H) Ventral sac; (I and J) Caudodorsal blind sac; (K and L) Caudoventral blind sac.

**Figure 2:**
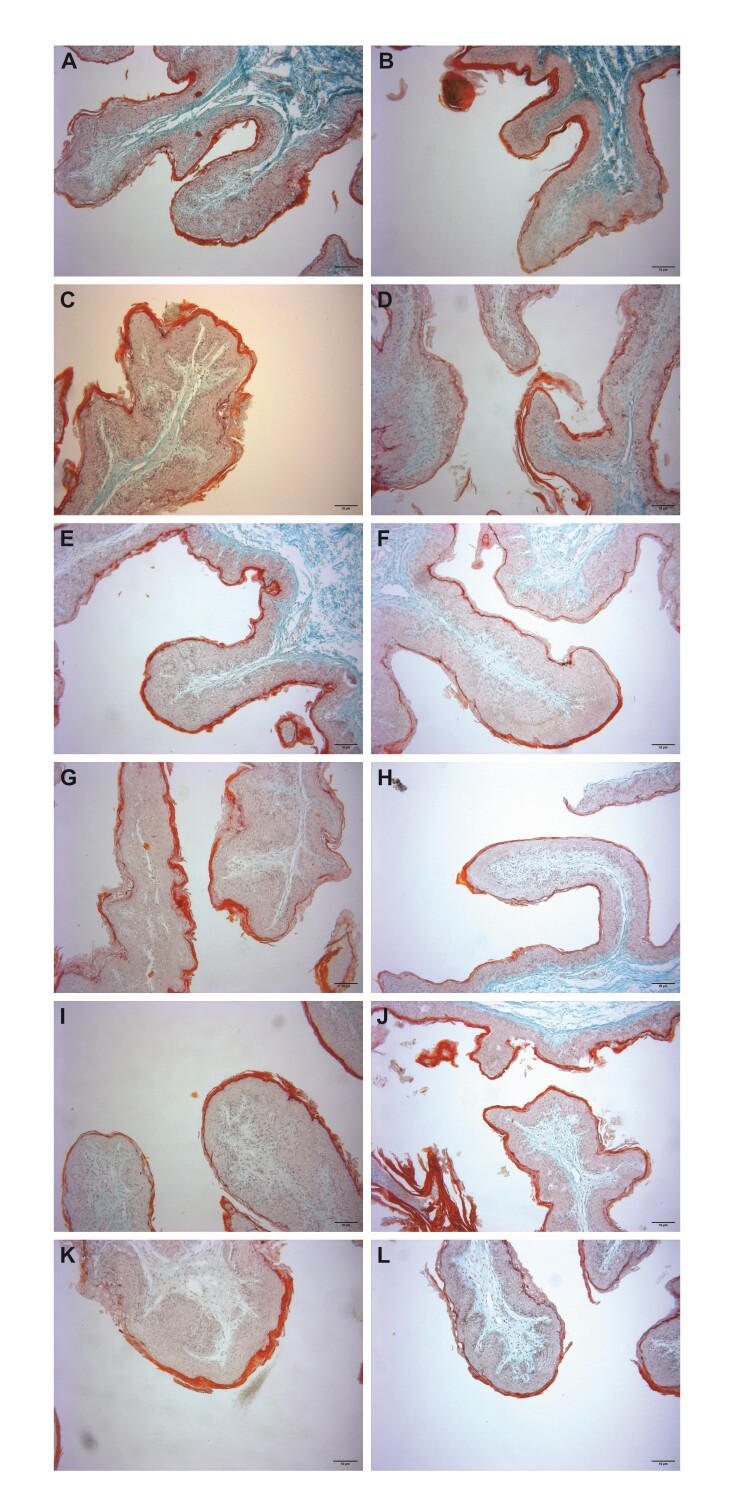
Optical Microscopy Images of the Ruminal Epithelium in Different Chambers Showing Papillae Development and the Keratin Layer (Masson-Goldner Trichrome). Left, group A; right, group B. 10×. (A and B) Atrium; (C and D) Recess; (E and F) Dorsal sac; (G and H) Ventral sac; (I and J) Caudodorsal blind sac; (K and L) Caudoventral blind sac.

**Figure 3:**
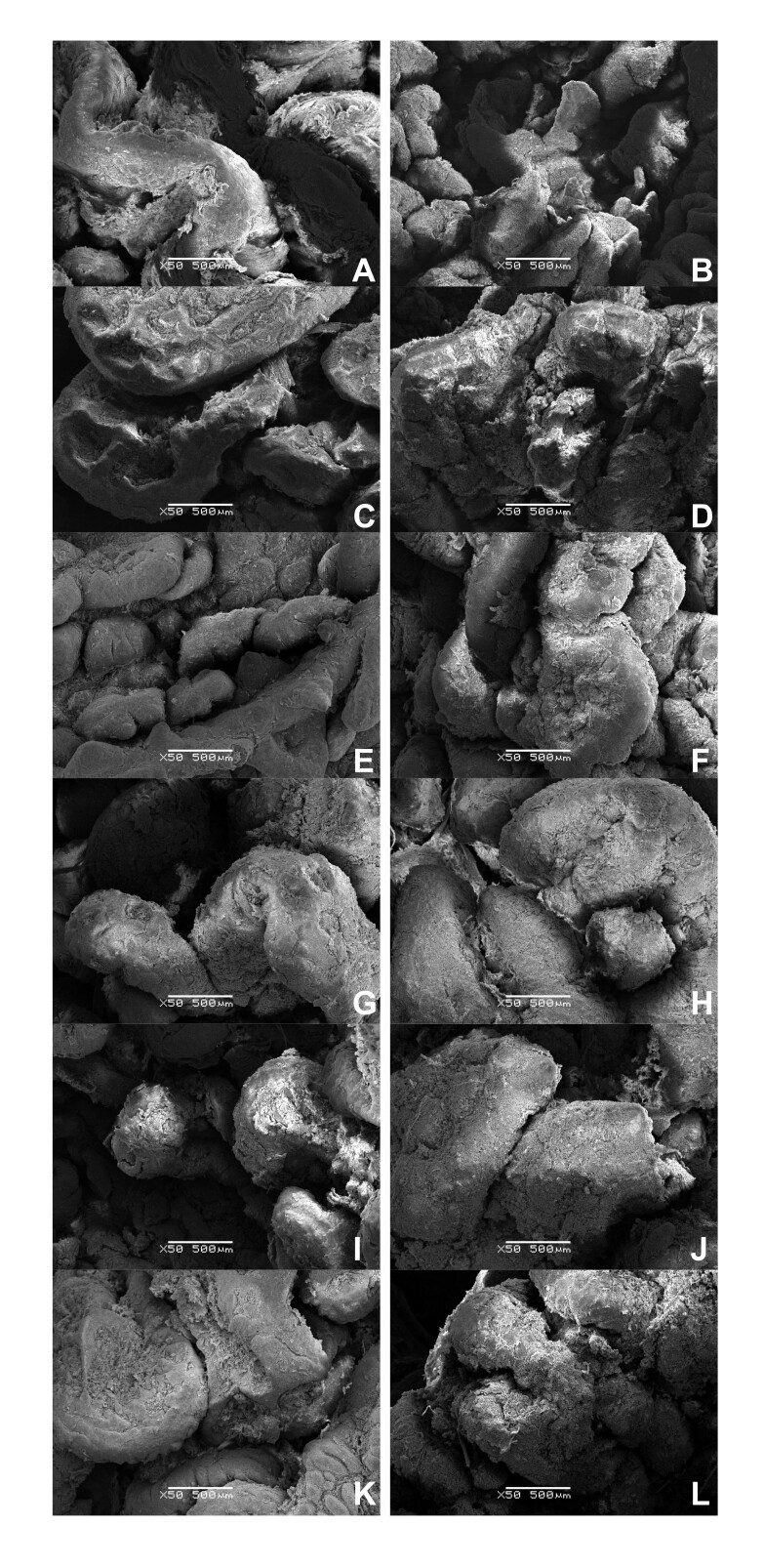
Scanning Microscope Images of the Ruminal Epithelium in Different Chambers Showing Papillae Development. Left, group A; right, group B. 50×. (A and B) Atrium; (C and D) Recess; (E and F) Dorsal sac; (G and H) Ventral sac; (I and J) Caudodorsal blind sac; (K and L) Caudoventral blind sac.

The ruminal mucosa of all animals was covered with typical papillae, except in the pillars (Figures 1, 2 and 3). Although not visible at first glance in the DS of the rumen, histological preparations revealed papillae development in this region (Figure 2). The papillae varied in shape and were of two main types: Filiform and foliate. In the DS and roof of the rumen, they were few in number, poorly developed, and often reduced to small tubercle-like structures (Figure 2). Each papilla consisted of a lamina of connective tissue from the mucosa, lined by stratified squamous epithelium, and contained small secondary papillae and adelomorphic extensions along the surfaces and edges (Figure 2).

**Figure 4:**
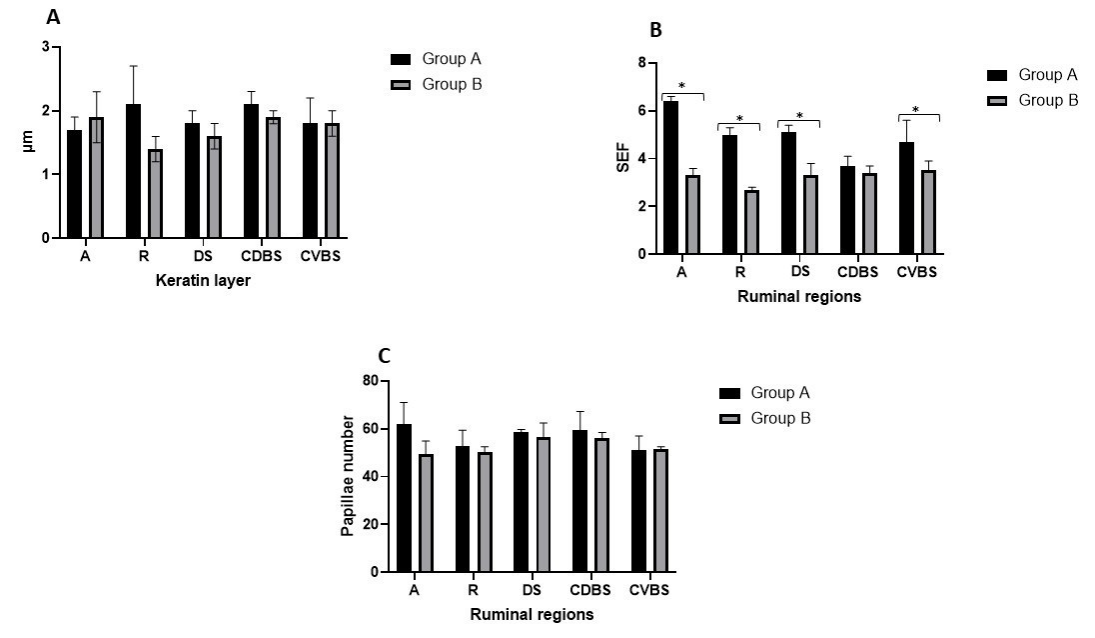
Comparison of Holstein Calves Fed 8 L/day of Milk Replacer with Starter (A) or Alfalfa Hay (B). Data are represented as the mean ± standard deviation (SD). (A) Comparison of the keratin layer thickness in different rumen chambers; (B) Surface enlargement factor (SEF) values in different rumen regions; (C) Numbers of papillae in different rumen regions. Regions: A, atrium; R, recess; DS, dorsal sac; VS, ventral sac; CDBS, caudodorsal blind sac; CVBS, caudoventral blind sac.

The ruminal mucosa appeared rough, pleated, and folded in the pillars. In contrast, papillae were abundant in the other regions (Figure 4). They were particularly numerous and tall in the atrium of calves in group A, progressively decreasing in height toward the blind sacs. In group B, papillae height was more uniform across the regions containing papillae.

The mean papilla height in the atrium was 5.25 mm (±1.31) in group A and 2.82 mm (±0.59) in group B. The number of papillae in each ruminal region is shown in Figure 4. The highest papillae density was observed in the atrium in group A and VS and CDBS in group B.

SEF measurements for each ruminal region and group comparisons are shown in Figure 4. SEF values were higher in group A than in group B, with statistically significant differences (*p* < 0.05) observed in all regions, except CDBS. The different *p* values for SEF in different regions were as follows: *p* < 0.0001, *p* < 0.0001, *p* = 0.0001, *p* = 0.2526, and *p* = 0.0218 in the atrium, recess, VS, CDBS, and caudoventral blind sac, respectively (not shown in the tables and figures). The highest SEF was recorded in the atrium in group A and blind sacs in group B. Figure 4 shows the keratin layer thickness in each rumen region in both animal groups.

## Discussion

In this study, Holstein calves fed a high MR allowance with starter feed (group A) exhibited greater papillae development and higher SEF values than those fed an MR supplemented with alfalfa hay (group B). These findings highlight solid feed composition as a decisive factor promoting epithelial growth, even under conditions of high liquid intake. The observed increase in papillae height, width, and density in starter-fed calves is consistent with previous reports that concentrate intake stimulates epithelial proliferation and mucosal mass, whereas exclusive milk feeding results in little or no ruminal papillae development (Brownlee, 1956; Weigand et al., 1975).

Concentrate feeding has been consistently associated with increased papillae density and height (Flatt et al., 1959; Rickard & Ternouth, 1965; Stobo et al., 1966), further confirming the strong impacts of dietary energy content and VFA production on ruminal morphology. However, this study involved only a limited number of calves and relatively short experimental period. Therefore, future studies with larger groups and longer monitoring periods are necessary to clarify the long-term implications for post-weaning performance and health.

The higher SEF in our starter-fed calves suggests an increased potential for VFA absorption. This is consistent with previous reports that fermentation products, particularly butyrate, act as potent epithelial proliferation stimulators (Diao et al., 2019; Tamate et al., 1962). The heterogeneous distribution of papillae across ruminal regions in both groups also supports the previously described pattern of digesta stratification and its influence on regional papillae development (Clauss et al., 2009a, 2009b).

Excessive starter intake compromises ruminal health. High-concentrate feeding is associated with reduced rumen motility, excessive papillary growth with keratinization, and decreased absorptive efficiency (Nocek & Kesler, 1980; Owens et al., 1993). In this study, keratin layer thickness did not differ significantly between the two groups, suggesting that the level of concentrate consumption, although sufficient to stimulate papillary growth, does not lead to pathological keratinization. This observation is consistent with the report of Beharka et al. (1998) that the balance between feed intake and fermentation end-products determines whether epithelial growth is adaptive or detrimental.

Recent studies have emphasized the complex relationship between diet composition and tissue development. Yohe et al. (2022) reported that high MR allowances combined with starch-rich starters promote visceral organ growth, particularly in the gastrointestinal tract, highlighting the role of energy supply in driving developmental responses. Similarly, Terler et al. (2023) reported that a combination of concentrate and hay of varying quality modulates rumen histology, digestive tract development, and subsequent slaughter performance. Consistently, our findings suggest that the nutritional composition and physical form of solid feed jointly shape rumen development, with energy-dense concentrates stimulating epithelial proliferation and fiber modulating the balance between functional maturation and structural growth.

The difference in the empty rumen weight between the two groups reflects more advanced papillae development in starter-fed calves than in hay-fed calves. In contrast, Khan et al. (2011) reported higher rumen-reticulum weight in hay-fed calves under a similar high milk allowance system. This discrepancy is possibly because the higher concentrate intake provided sufficient energy to support epithelial growth, thereby reducing the expected advantage of forage in stimulating physical development in this study. This interpretation also supports the hypotheses of Cozzi et al. (2002) and Suárez et al. (2006, 2007) that fiber in concentrate-rich diets mitigates ruminal dysfunction, although dietary energy density remains the primary driver of epithelial development.

Diet composition during the pre-weaning phase profoundly impacts rumen morphological development. Although accelerated rearing with a high milk allowance ensures rapid body growth (Brown et al., 2005a, 2005b; Vieira et al., 2008), the type of solid feed determines the extent of epithelial maturation. In this study, starter feeding resulted in greater papillae development and a larger absorptive surface area than forage alone, suggesting a more efficient post-weaning adaptation to ruminant digestion in starter-fed calves.

## Conclusion

In conclusion, this study found that high MR allowances combined with starter feed promoted greater ruminal papillae development than forage alone during the pre-weaning phase. Despite increased milk intake, the type of solid feed proved to be decisive for epithelial maturation, with starter feed stimulating greater papillae development and larger surface enlargement. These findings highlight the importance of providing concentrate-rich starter from the first weeks of life, even under accelerated milk-feeding programs, as it facilitates a smoother transition to solid diets at weaning.

This study has some limitations. The relatively small sample size (n = 20) and short experimental duration (10 weeks) limit the generalizability of our findings to broader production systems. Moreover, the number of animals was constrained by ethical and logistical factors inherent to terminal studies involving postmortem tissue analysis. Nevertheless, our experimental design and statistical analyses successfully detected significant differences in key morphometric parameters, supporting the robustness of our conclusions. Our results clearly demonstrated enhanced rumen epithelial development in starter-fed calves; however, the potential long-term implications, such as post-weaning adaptation, health, and productivity, remain unknown. Therefore, future studies with larger populations and longer follow-up periods should confirm the persistence of these effects to better understand their physiological relevance during the transition to ruminant digestion.
